# A patient journey mapping study of lived experiences during platelet-rich fibrin gel therapy for venous leg ulcers

**DOI:** 10.3389/fmed.2025.1652687

**Published:** 2026-01-09

**Authors:** Feifei Cui, Zhen Xu, Rongting Wang, Shuainan Chen, Qiaodan Hu, Haiying Wu

**Affiliations:** 1 Department of Nursing, The Affiliated Dongyang People’s Hospital, Wenzhou Medical University, Dongyang, Zhejiang, China; 2 Department of Central Blood Bank, The Affiliated Dongyang People’s Hospital, Wenzhou Medical University, Dongyang, Zhejiang, China

**Keywords:** active venous leg ulcers, patient journey mapping, platelet-rich fibrin gel, psychological-cognitive model, psychologicaldynamic, qualitative research, quality of life, therapeutic experience

## Abstract

**Background:**

Active venous leg ulcers (VLUs), characterized by high recurrence and complexity of treatment, place patients under a dual physiological and psychological stress. Platelet-rich fibrin (PRF) shows biological therapeutic potential, but current research predominantly focuses on its clinical efficacy through quantitative measures such as wound healing rates, leaving a significant gap in understanding the holistic therapeutic experience and multidimensional psychological-cognitive journey of patients undergoing this novel treatment. This gap limits the development of patient-centered care strategies that could optimize both biological and psychosocial outcomes.

**Objective and methods:**

This study aimed to identify key stages and touchpoints where patient needs, distress, and decision-making are most pronounced during PRF gel therapy, by employing patient journey mapping to visualize and analyze the holistic treatment experience. A descriptive phenomenology study using semi-structured interviews was conducted in 13 active VLU patients from June to December 2024. The data were analyzed using the seven-step Colaizzi method to identify themes and to construct a dynamic journey model that integrates cognition, experience, and emotions.

**Results:**

The analysis revealed four core themes: pre-treatment cognitive dissonance and hope; treatment-phase challenges; post-treatment biopsychosocial shifts; and dynamic anxiety-hope oscillation.

**Conclusion:**

Patients reported that PRF gel therapy, within the context of comprehensive wound care, was associated with enhanced wound healing and improved psychological wellbeing. The proposed patient journey map translates these lived experiences into a structured framework, offering actionable insights for implementing patient-centered care and optimizing management strategies for refractory VLUs.

**Implications:**

Clinically, this framework aids in identifying high-need phases of the treatment journey and provides a basis for tailoring patient education and support, ultimately helping to improve both clinical workflows and patient engagement in PRF-based wound management.

## 1 Introduction

### 1.1 Epidemiology, pathophysiology, and treatment challenges of active venous leg ulcers

Active venous leg ulcers (VLUs) represent the terminal stage (CEAP C6) of chronic venous insufficiency, characterized by full-thickness skin defects resulting from impaired venous return in the lower limbs (1–4). These ulcers are highly refractory and prone to recurrence (5–7). Globally, VLUs affect 1.5–3% of adults aged over 65 years (3, 5, 8), incurring annual direct medical costs exceeding $15 billion (9). A qualitative meta-synthesis confirms that emotional suffering–including depression, anxiety, fear, and disappointment—is pervasive among VLU patients and intrinsically linked to wound-related physical discomfort and social limitations (8, 10). This psychological burden, exacerbated by treatment uncertainty and life restrictions, severely compromises quality of life (9, 11, 12). Such comorbidity is not unique to VLUs but reflects a broader pattern in chronic dermatological conditions; for example, patients with psoriasis show markedly elevated rates of anxiety, depression, and suicidal ideation compared to the general population (13). This consistent pattern underscores the critical and generalizable link between chronic skin disease and impaired mental health.

Traditional treatments for venous leg ulcers (VLUs)—including compression therapy, debridement, and surgery—face significant limitations in both efficacy and adherence. These modalities often inadequately address the multifactorial pathogenesis of VLUs (14, 15), with compression therapy achieving healing in only 40–60% of patients after 24 weeks (4, 7). Furthermore, adherence remains below 50% (16, 17) due to treatment-related discomfort, restricted mobility, economic burden (4), and psychological resistance (14). This combination of modest efficacy and poor adherence often results in a cycle of non-healing, recurrence, and patient frustration, prompting many patients to seek more acceptable and effective conservative alternatives (18).

These limitations highlight the need for novel therapies that improve both biological and experiential outcomes. Platelet-rich fibrin (PRF) gel has emerged as a promising biological treatment, yet its impact on the patient experience—including cognitive, emotional, behavioral, and contextual dimensions throughout treatment—remains systematically unexamined. This identified gap directly motivates the present study. While platelet-rich fibrin (PRF) gel shows biological promise, focusing solely on its efficacy while neglecting how patients perceive, accept, and persist with treatment in real clinical settings risks undermining its potential value due to experiential barriers. Therefore, this study aims to systematically trace and visualize the multidimensional experiences and dynamic decision-making of patients during PRF therapy by integrating qualitative interviews with patient journey mapping. In doing so, it seeks to bridge the divide between “efficacy evidence” and “lived reality,” thereby providing an empirical foundation for developing a truly patient-centered clinical pathway for PRF implementation.

### 1.2 Platelet-rich fibrin gel: biological potential and the uncharted dimension of patient experience

Platelet-rich fibrin (PRF) gel has gained increasing attention as a novel autologous biological material for treating venous leg ulcers in recent years (19–23). Evidence from meta-analyses and randomized controlled trials supports the potential of PRF to enhance healing in chronic wounds. A meta-analysis by Chen et al. focusing on hard-to-heal skin ulcers found that the PRF group had a 48% higher absolute rate of complete healing at 4 weeks compared to control (RD = 0.48, 95% CI 0.31–0.66), with the benefit remaining significant at the end of follow-up (RD = 0.17, 95% CI 0.08–0.26) (24). Other studies have similarly reported faster wound area reduction and improved healing rates in VLU patients treated with PRF gel compared to conventional therapy (19, 20).

The majority of studies employ randomized controlled trials (RCTs) to quantify wound healing rates (25–29), while few systematically examine patients’ internal experiences, psychological-cognitive shifts, decision-making challenges, and adherence during PRF therapy (30). Although some qualitative studies assess patient attitudes via satisfaction scales (30, 31), they offer only fragmented perspectives and fail to fully analyze the complex psychosocial interactions inherent in treatment (32). Moreover, existing research often isolates PRF’s biological effects from patients’ psychological states, neglecting their dynamic interplay. Key questions remain unanswered, for instance, how wound severity or healing rate influences treatment trust, or how clinician–patient communication shapes pain perception.

### 1.3 Theoretical framework and applicability of patient journey mapping

Patient journey mapping (PJM) is a visual tool that systematically identifies care process pain points and intervention opportunities by integrating data on patient behavioral trajectories, emotional changes, and interactions with the healthcare system throughout the treatment journey (33–35). Its core components include a temporal axis (depicting the continuum from symptom onset to long-term follow-up), multidimensional data layers (e.g., physiological indicators, psychological status, and social support), and key interaction nodes (such as clinician–patient communication and inter-departmental coordination).

This framework is particularly suitable for investigating the complex experience of VLU patients undergoing PRF gel therapy. As an emerging biological treatment involving multi-departmental collaboration, the therapeutic experience of PRF is closely linked to patients’ psychological cognition, treatment expectations, and perception of the care process. By constructing a PRF treatment journey map, it is possible to dynamically trace the cognitive evolution and emotional fluctuations of patients through the decision-making, treatment, and follow-up phases. This approach can systematically reveal the interactive mechanisms between “perceived biological efficacy” and “psychosocial adaptation,” thereby addressing the current research gap in the dynamic and holistic understanding of the experiential dimension. Consequently, it provides structured empirical evidence for optimizing patient-centered PRF clinical pathways.

### 1.4 Knowledge gaps and study aim

Although PRF demonstrates biological therapeutic potential, no study has systematically explored the holistic, multidimensional, and dynamic experiences of patients during its administration, leaving a significant knowledge–practice gap. Specifically, this study aims to address the following three lacunae: (1) the unique psychological-cognitive journey of patients undergoing PRF therapy; (2) the dynamic interaction between perceived biological efficacy and psychological state; and (3) the contextualized barriers (e.g., procedural, economic, and communicative) influencing PRF treatment adoption and adherence.

To this end, we innovatively integrate descriptive phenomenology with patient journey mapping to systematically trace and visualize patients’ experiential trajectories during PRF therapy, thereby contributing to the development of a more humanistic and clinically effective chronic wound management pathway.

## 2 Materials and methods

### 2.1 Study design and recruitment of participants

This descriptive qualitative study employed a purposive sampling strategy to recruit participants with active VLU from a tertiary hospital in Zhejiang Province, China, between June and December 2024. The aim was to enroll individuals who could provide rich, in-depth insights into the experience of PRF gel therapy.

Inclusion criteria were as follows: (a) age >18 years; (b) active VLU at CEAP clinical class C6 (full-thickness skin defect); (c) ulcer duration >4 weeks and size 4–20 cm^2^; (d) ultrasound-confirmed venous insufficiency; (e) clean wound bed without systemic infection (C-reactive protein <10 mg/L, body temperature <37.3 °C); (f) ankle-brachial index >0.8 and tolerance to compression therapy; and (g) intact cognitive function with the ability to provide written informed consent.

Exclusion criteria included the following: (a) thrombocytopenia (<100 × 10^9^/L) or current use of anticoagulants; (b) severe peripheral arterial disease (ankle-brachial index <0.6); (c) immunodeficiency or active severe infection (e.g., necrotizing fasciitis).

Recruitment continued until thematic saturation was achieved, meaning that no new themes or insights relevant to the research questions emerged from subsequent interviews (36). Saturation was confirmed after the analysis of the 11th interview, and two further interviews were conducted for confirmatory purposes, resulting in a final sample of 13 participants. All participants were recruited from and treated within the specialized wound care clinic of the aforementioned tertiary hospital. To ensure consistency in the clinical protocol, all patients continued to receive standardized compression therapy as the foundational wound management throughout the study period. The PRF gel preparation, application, and subsequent wound dressing procedures were performed by the same team of certified wound care specialists according to a unified hospital protocol. This approach minimized variability in clinical care delivery and allowed the study to focus more distinctly on the patient experience of the PRF treatment itself. This study received ethical approval from the Clinical Research Ethics Committee of Dongyang Hospital of the Wenzhou Medical University (Approval No.: 2023-YX-019). All participants provided written informed consent after receiving a complete explanation of the study.

### 2.2 Research methods

**Figure 1:**
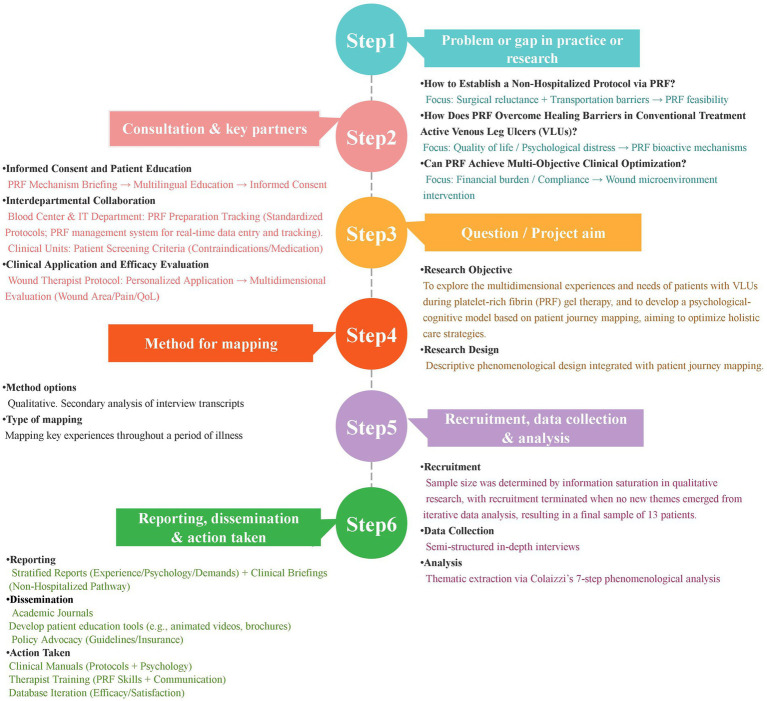
Patient journey mapping for PRF gel therapy in active venous leg ulcers (VLUs). This flow chart illustrates an integrated framework for PRF gel therapy for VLUs structured by patient journey mapping.

The study is a qualitative research design that innovatively integrates two qualitative methodological frameworks: descriptive phenomenology and patient journey mapping (PJM). A visual overview of the integrated methodological steps, from study design to dissemination, is provided in Figure 1. The research team followed the methodological steps to construct the patient journey map (34, 35, 37–39), including desktop research on clinical protocols and practices, combined with participant observation methodology to illustrate the journey framework, followed by interviews with patients to further refine the details of the journey and suggest innovations and improvements, and finally by an evaluation of the map with stakeholders (physicians, nurses, patients, and families).

#### 2.2.1 Integrated methodological framework: phenomenology and patient journey mapping

Descriptive phenomenology provided the philosophical underpinning to deeply explore the lived experience and essence of patients’ perceptions. Patient journey mapping (PJM) was then employed as a structured visual-analytical tool to organize these rich phenomenological data chronologically and across multiple dimensions (cognitive, emotional, behavioral, and interactional). PJM translated the depth of phenomenology into an actionable, clinical-care framework.

#### 2.2.2 Desktop study

The research team reviewed relevant institutional documents to establish the standard clinical pathway. This included the hospital’s clinical protocol for PRF preparation and application in chronic wounds, standard operating procedures for the wound care clinic, and sample patient information leaflets regarding PRF therapy. This review informed the initial drafting of the treatment timeline and key procedural nodes for the patient journey map.

#### 2.2.3 Participant observation

Researchers observed three patients during the clinical workflow (outpatient evaluation and wound care: preparation, application, and follow-up), documenting critical touch points (e.g., dressing changes, interdepartmental transfers, and communication between clinician and patient), and non-verbal behaviors (e.g., facial expressions and body language). Based on field observations, the research team structured the analysis around four predefined analytical dimensions: (1) cognitive State (e.g., patient’s understanding of PRF), (2), decision-making (e.g., moments of consent or hesitation), (3) emotional dynamics (e.g., observed anxiety or relief), and (4) multidimensional interaction (e.g., communication with staff and logistical challenges). These dimensions provided a consistent framework for coding observational notes and, later, for organizing the qualitative interview data within the patient journey map.

#### 2.2.4 Semi-structured interviews

An interview guide was developed based on a literature review, expert consultation (three vascular surgeons and two wound specialists), and pilot interviews (*n* = 3). The guide contained six key questions: (1) ‘What experience of PRF treatment has left you most impressed?’; (2) ‘What did you know before PRF treatment and what did you expect?’; (3) ‘Can you please describe in detail your experiences during the PRF treatment process?’; (4) ‘How would you assess the effectiveness of PRF treatment?’; (5) ‘How would you assess the impact of PRF treatment on your daily life?’; and (6) ‘What would you advise other patients who are about to be treated with PRF?’

Before the interview, the researcher contacted the participants to establish a rapport and explain their role and the purpose of the study. After obtaining their consent, the participants signed the informed consent form. Semi-structured, face-to-face interviews were then conducted in the outpatient clinic consultation room (40–60 min), exploring pre-treatment cognition, in-treatment experiences, and post-treatment adjustment. Interviews were audio recordings with consent, and non-verbal behaviors (e.g., emotional changes and gestures) were documented. This study is based on the core performers reporting standards (COREQ), see Supplementary Table 1.

The study adopted Colaizzi’s phenomenological method for data analysis (40). The analysis followed these core steps to ensure an in-depth and systematic interpretation: (1) immersive familiarization with the data and extraction of significant statements; (2) formulation of meanings and development of themes/sub-themes through clustering of meaning units; (3) integration of themes into a structured description aligned with the patient journey stages; and (4) validation through member checking. To enhance the transparency and rigor of the thematic development (Steps 1–2), two researchers (SC and QH) independently coded the first three interview transcripts to develop a preliminary codebook. Discrepancies were discussed and resolved through consensus meetings involving a third researcher (FC). This collaborative codebook was then applied to the remaining transcripts. Given the interpretive nature of phenomenological analysis, which prioritizes depth of understanding and consensus over frequency, formal inter-coder reliability statistics (e.g., Cohen’s kappa) were not calculated. An illustrative outline of the coding structure, showing the progression from meaning units to sub-themes and themes, is provided in Supplementary Table 1.

For member checking (Step 4), the preliminary thematic structure and a summary description were returned to three participants (Patients 2, 7, and 11), who were purposefully selected to represent variation in age and ulcer duration. All three confirmed that the analysis accurately reflected their experiences. One participant (Patient 7) emphasized the salience of concerns regarding scar esthetics; this feedback led to a more nuanced presentation of the sub-theme ‘concerns about scarring and cosmetic outcomes’ in the Results section.

The rigor of the data in this study is based on the empirical criteria of Lincoln and Guba, as follows (41): (1) Credibility: The researcher has received professional training and hands-on experience in qualitative research. Before conducting in-depth interviews, the researcher was already familiar with the participants through their role as a wound care nurse and had developed a therapeutic relationship with them. During the interview process, any questions that were not understood were rephrased or clarified to ensure participants’ responses were accurately captured, thereby enhancing data credibility; (2) Reliability: The study was conducted by the researcher using sampling, direct care, and semi-structured one-on-one interviews. All interviews were recorded with a tape recorder and transcribed verbatim. Interview excerpts were used to capture participants’ ideas, situations, and researcher–participant interactions; (3) Confirmability: To enhance the objectivity of interpretation, the researcher employed bracketing throughout the research process. This involved maintaining a reflective journal to consciously identify and set aside prior assumptions and clinical expectations regarding PRF treatment, thereby focusing the analysis on the participants’ described lived experiences. Additionally, three participants (Patients 2, 7, and 11) were invited to review the preliminary thematic analysis (member checking), confirming that the findings accurately reflected their experiences and thereby further mitigating interpretive bias; and (4) Transferability: Transferability was supported by clearly describing the participant selection criteria and presenting the complete wound treatment experience of the participants. Their journey and feelings were portrayed in a realistic manner, so that the research results of the survey can be used as a reference for similar experiences.

#### 2.2.5 Visualization of the patient journey

The patient journey map was developed through a synthesis of observational notes and semi-structured interview data, informed by a two-stage analytical process. First, interview transcripts underwent full phenomenological analysis using Colaizzi’s method to identify significant statements, formulate meanings, and cluster these into themes. To visually structure these thematic findings within a temporal and multidimensional framework, we then transposed and mapped the resulting themes and sub-themes onto the two primary axes of the journey map: the chronological timeline of treatment phases and the predefined analytical dimensions (e.g., cognitive state and emotional dynamics). This integrative synthesis step-organizing phenomenological insights into a structured visual model did not alter the original analysis but enhanced its presentation to highlight dynamic relationships across the treatment journey. Rigor was ensured by maintaining traceability between the map’s visual elements and the underlying qualitative data, validated through team consensus and member checking.

The specific procedural steps for this synthesis were as follows: Within 24 h of each interview, verbatim transcripts were produced. Significant statements were extracted and coded into meaning units aligned with a four-phase cognitive model (representing the treatment timeline) and three analytical dimensions (e.g., cognitive, emotional, and behavioral). To enrich the visualization, direct patient quotes were embedded at relevant nodes. Furthermore, the patient journey map was further enriched by integrating standardized patient-reported outcome measures, specifically, Visual Analog Scale (VAS) pain scores and Generalized Anxiety Disorder-7 (GAD-7) questionnaire scores, as descriptive, supplementary data layers. These quantitative descriptors provided a concurrent, longitudinal overview of patients’ physical and emotional states at key timepoints throughout the treatment journey. This integration served to add contextual depth and an additional dimension to the qualitative narrative, thereby contributing to a more comprehensive and multi-faceted visual representation of the patient experience. It is important to note that these scores were used for descriptive illustration within the qualitative framework and did not constitute formal quantitative analysis.

### 2.3 PRF gel preparation and treatment protocol

#### 2.3.1 Wound bed preparation and co-interventions

Before the PRF application, all wounds underwent standardized bed preparation based on TIME principles. Crucially, all patients continued a consistent co-intervention regimen throughout the study: oral venoactive medications (as prescribed by vascular surgeons), multilayer compression therapy, and standardized patient education on limb elevation, skin care, and calf muscle exercises. Patients completed the GAD-7 and a VAS for wound pain (0–10) at three timepoints: baseline (pre-treatment), before each weekly PRF application, and at the final follow-up. Scores were recorded in their electronic health records and extracted for this study to enrich the journey map descriptively. No data were missing for these timepoints.

#### 2.3.2 PRF preparation, application, and dressing protocol

Peripheral venous blood (approximately 5 mL per required gel unit) was collected into sterile glass tubes without an anticoagulant. Centrifugation was performed within 60–90 s post-collection using a fixed-angle centrifuge at 3000 rpm for 10 min (relative centrifugal force approximately 400 *g* at a radius of 15 cm). After centrifugation, the middle fibrin clot layer (PRF gel) was separated from the acellular plasma and red blood cell layers using sterile technique.

After wound bed preparation, the PRF gel was applied directly to the ulcer bed. It was covered with alginate dressing, followed by an absorbent secondary dressing, and secured with a multilayer compression bandage system. This dressing change, including PRF re-application, was repeated weekly until healing criteria were met or for a predefined maximum period. Wound area was assessed weekly using the “clock face” method (measuring the longest length, perpendicular width, and depth). Digital photographs and patient-reported outcomes (VAS pain and GAD-7 anxiety) were collected at each visit to monitor progress.

### 2.4 Researcher reflexivity

The primary researchers involved in data collection and analysis are wound-care nurses within the study clinic. This insider’ clinician-researcher position facilitated trust and clinical understanding but also introduced potential biases. We recognized the risk of social desirability bias (patients providing answers they believed clinicians wanted to hear) and the influence of our clinical assumptions on data interpretation. To mitigate these effects, we implemented several strategies: (1) Reflexive journaling was maintained throughout the study to consciously identify and bracket pre-existing clinical assumptions; (2) Interview questions were designed to be open-ended and non-leading to encourage authentic narratives; (3) A researcher from a non-clinical department (the blood bank, ZX) participated in data analysis to provide an external perspective on the thematic interpretation; and (4) Member checking with participants was employed to verify that our analysis resonated with their lived experience, not solely our clinical reading of it.

## 3 Results

Thirteen patients with active VLUs (eight men and five women) participated. The median age was 67 years (range: 33–87), with 69.2% (*n* = 9) aged over 50 years. Ulcers were primarily located on the gaiter area (ankle and lower tibia). There was considerable clinical heterogeneity: ulcer duration ranged widely from 22 to 168 days, and wound areas varied from 2.99 to 55.00 cm^2^. The mean venous clinical severity score (VCSS) was 15.00 ± 2.89 (range: 10–19). Common comorbidities included hypertension (*n* = 7), diabetes mellitus (*n* = 4), and a history of venous thrombosis (*n* = 3). Social support during clinic visits also varied; while most were accompanied by family members (spouse and children), a subset of patients attended appointments independently. Detailed demographic and clinical characteristics are summarized in Table 1. Descriptive wound tracking revealed a positive trend: 11 of 13 participants achieved >50% area reduction within the initial period, and 11 attained complete epithelialization by the study endpoint. The descriptive summary of wound healing trends is presented in Table 2.

**Table 1:** General information about the patients who participated in the interviews.

Code	Gender	Age (years)	Site	Duration (days)	Area (cm^2^)	Venous Function Score	Complication	Drink	Smoking	Accompanying personnel
P1	Female	71	Anterior tibia	127	55.00	17	Diabetes, hypertension	No	No	Spouse/son
P2	Male	35	Ankle	40	14.00	12	Deep vein thrombosis	No	Yes	Alone
P3	Male	45	Anterior tibia, ankle	118	48.75 15.00	19	Diabetes, hypertension, venous thrombosis	Yes	Yes	Alone
P4	Male	68	Ankle	31	4.40	16	Hip replacement	No	No	Alone
P5	Female	75	Anterior tibia	22	2.99	15	Hypertension	No	No	Alone/son-in-law
P6	Male	33	Anterior tibia	95	7.88	10	Autoimmune disease	No	No	Alone
P7	Male	72	Anterior tibia	96	3.00	12	Diabetes	Yes	No	Alone/daughter
P8	Female	51	Anterior tibia, ankle	31	11.00 6.25	19	Hypertension	No	No	Spouse
P9	Female	76	Anterior tibia	168	33.00	18	Polycythaemia	No	No	Daughter/daughter-in-law
P10	Male	87	Anterior tibia	64	9.00	14	Hypotension	No	No	Son
P11	Male	52	Anterior tibia	46	22.50	15	Diabetes, hypertension	Yes	No	Spouse
P12	Male	35	Ankle	52	16.50	12	Venous thrombosis	No	Yes	Alone
P13	Female	67	Ankle	69	9.00	16	Hypertension	No	No	Alone/son

**Table 2:** Descriptive summary of wound healing trends.

Code	Initial area (cm2)	3 week area (cm2)	8 week area (cm2)	12 week area (cm2)	Healing status at study end	Healing rate (at 3 week)
P1	55.00	22.05	13.86	3.75	>75% reduction	59.91%
P2	14.00	4.25	0	/	Healed	69.64%
P3	48.75 15.00	11.2 4.5	1.2	/	Healed	77.03%
P4	4.40	1.2	0	/	Healed	72.73%
P5	2.99	0		/	Healed	100.00%
P6	7.88	1.44	0	/	Healed	81.73%
P7	3.00	1.84	0	/	Healed	38.67%
P8	11.00 6.25	1.25 0.8	0	/	Healed	88.64%
P9	33.00	26.4	9.9	2.8	>75% reduction	20.00%
P10	9.00	3.5	1.04	0	Healed	61.11%
P11	22.50	9.6	3.28	0	Healed	57.33%
P12	16.50	3	0	/	Healed	81.82%
P13	9.00	3.4	0	/	Healed	62.22%

Healing Status categories: “Healed” (100% epithelialization), “>75% reduction,” “50–75% reduction,” “<50% reduction”. For participants P3 and P8, who had wounds at two distinct anatomical sites, the 3-week healing rate was calculated based on the site with the larger initial wound area.

**Figure 2:**
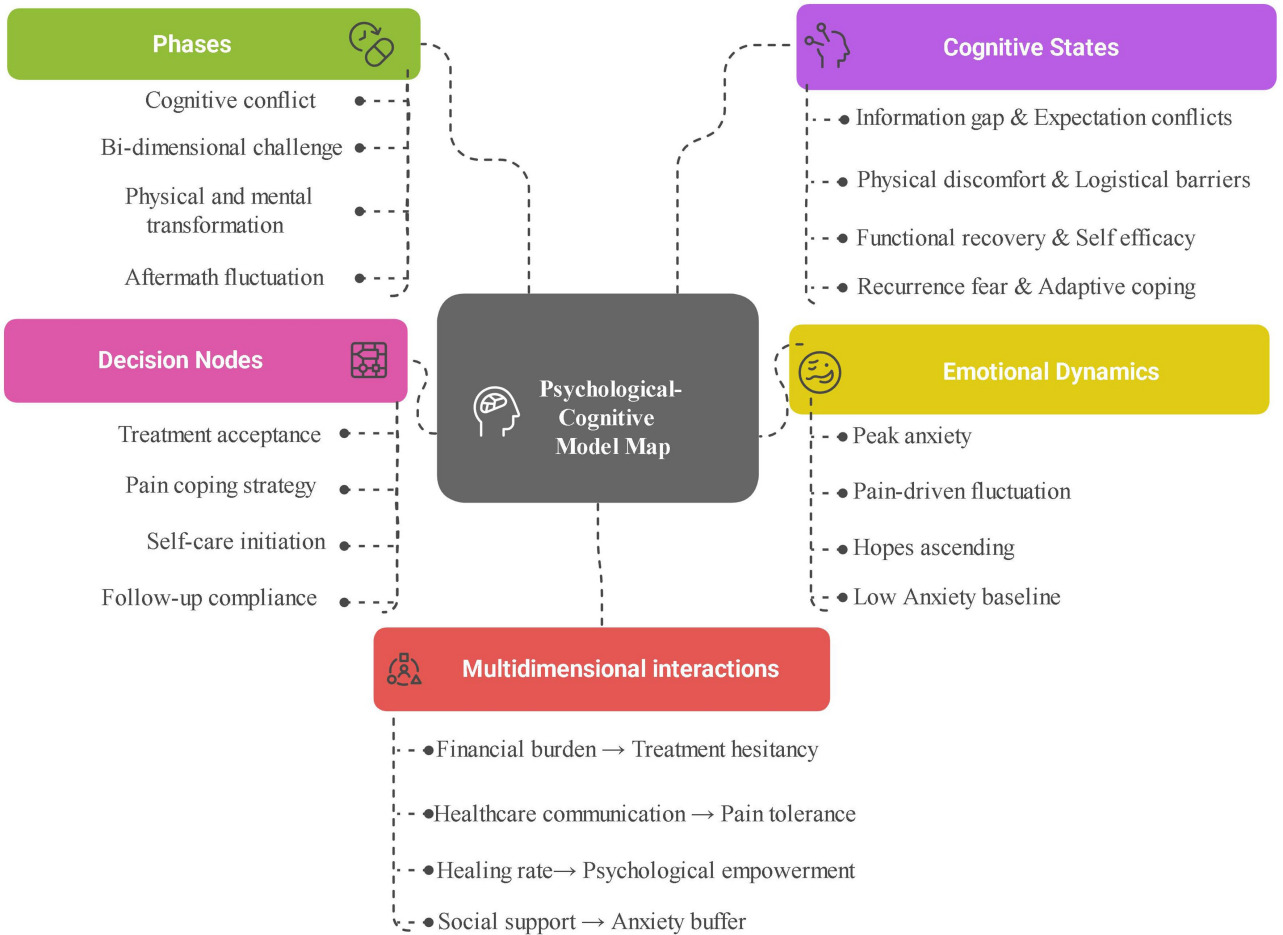
Initial psychological-cognitive model for patients undergoing PRF gel therapy. This schematic map conceptualizes the dynamic mental-cognitive processes in patients with VLU who are receiving PRF gel therapy.

Building a patient journey map usually involves a horizontal axis (time axis) and a vertical axis (task axis) (42). In this study, the horizontal axis of the psycho-cognitive journey map of PRF applied to patients with active leg ulcers was divided into four phases: cognitive conflict, dual-dimensional challenge, physical and mental transformation, and aftermath fluctuation. The vertical axis includes the cognitive state, decision-making, emotional dynamics, and multidimensional interaction in the patients’ therapeutic journey according to the objectives of this study. Based on a narrative framework, a detailed exploration of the content of the patient experience in different dimensions at each stage, an analysis of four themes, eight sub-themes, and 16 items, the relationship between these themes, the chronological stages of treatment, and the core analytical dimensions (cognitive state, decision-making, emotional dynamics, and multidimensional interaction) is synthesized in Figure 2.

**Figure 3:**
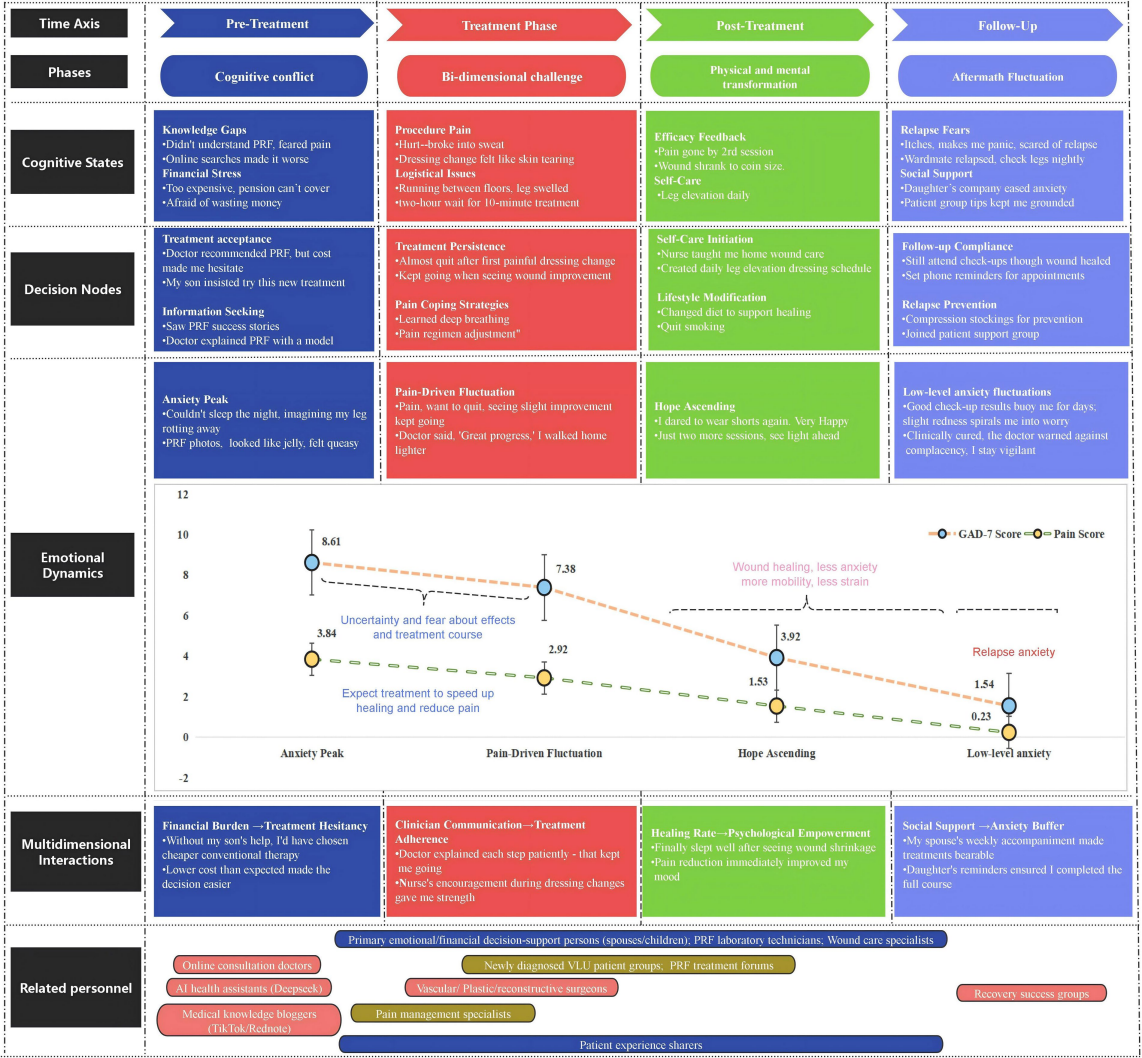
Temporal psycho-cognitive journey mapping of PRF therapy for venous leg ulcers. This integrated model defines the dynamic interaction between clinical progression, psychological status, and behavioral decision making in patients with venous leg ulcers treated with PRF gel. Longitudinal thematic analysis using Colaizzi’s method. The GAD-7 Anxiety Scale (0–21) and the VAS Pain Scale (0–10) are used to measure anxiety and pain.

A detailed, annotated patient journey map, integrating qualitative narrative data with descriptive patient-reported outcome scores across the treatment timeline, is presented in Figure 3. This figure provides a comprehensive visual narrative of the interconnected experiences described in the following sections.

### 3.1 Cognitive dissonance and multidimensional expectations before treatment

#### 3.1.1 Limited access to information

Before treatment, patients’ knowledge of PRF treatment was generally limited. They were primarily introduced to the PRF through healthcare professionals and promotional leaflets distributed by hospitals. Although patients received information about the general principles and expected effects of the treatment, their overall knowledge remained limited, especially regarding the underlying biological mechanisms, leading to confusion about the treatment to be given.

*“Not much is known about it, to be honest, my doctor has told me that it is a new method that might help me to heal, but I'm not too sure exactly how it works." (P10)*

*“I found out about the treatment from my doctor and the nurse in the dressing room. They explained how the treatment works and what to expect, but I still don't quite understand it.” (P3)*

*“New conservative treatment as opposed to surgery, and having read about some successful cases, I still didn't know what to think.” (P13)*

#### 3.1.2 Internal struggle between expectations and concerns

**Figure 4:**
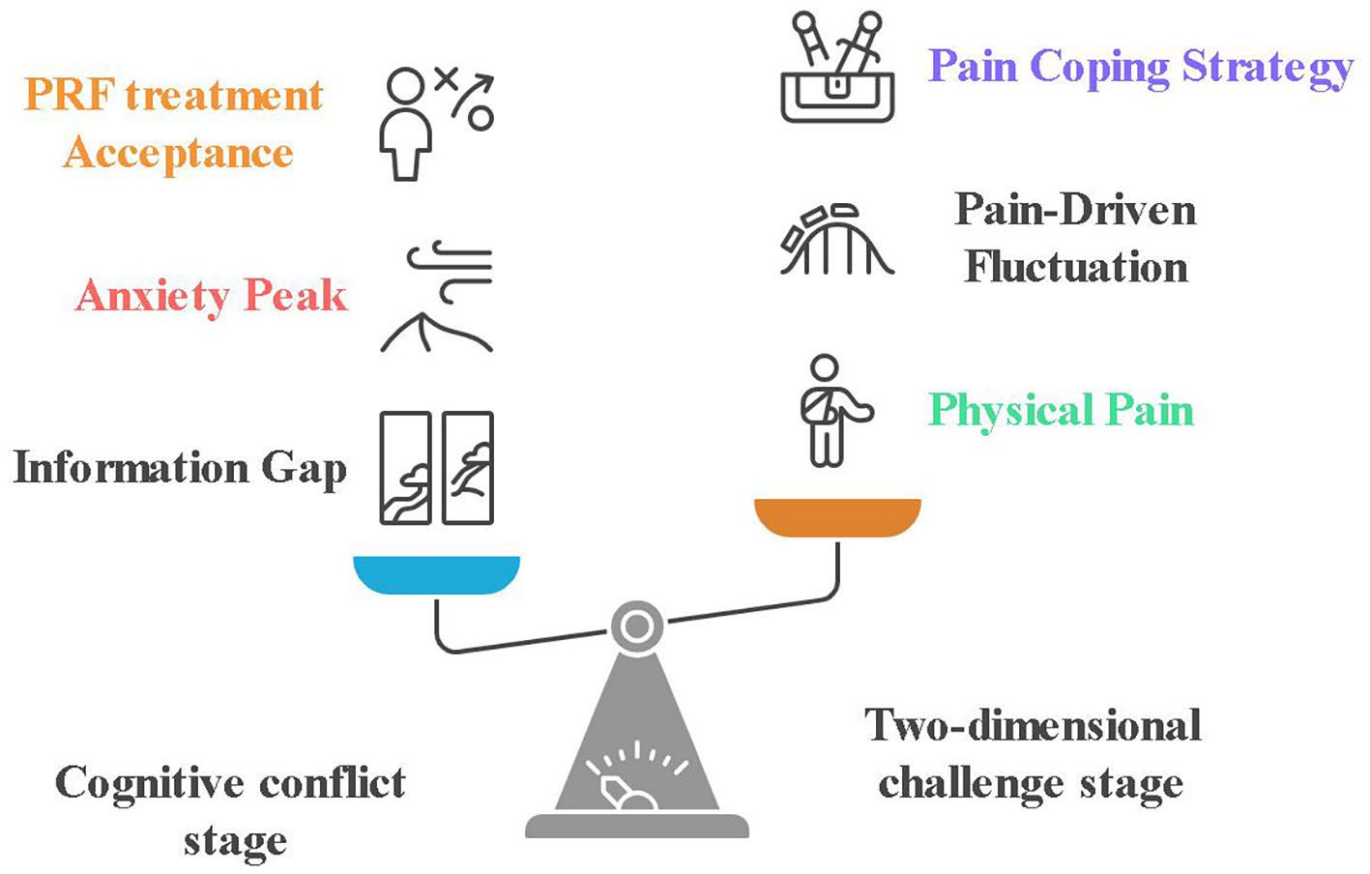
Dual axis framework for decision-making in VLU patients. The model structurally validates the patient’s experience of being caught between hope and despair—where desire for faster healing meets cost–benefit considerations, culminating in the observed state of limbo. The dual axis model in Figure 4 identifies anxiety peaks as emergent features of unmet expectations of recovery colliding with unresolved practical obstacles. This dual reinforcement of financial burdens and reluctance to treat explains why patients who expect rapid relief of pain may also delay the adoption of PRF. Notably, the anxiety peak condition reflects their conflicting desire for rapid healing with unresolved financial and technical concerns.

Patients generally reported having chronic wounds that were costly to manage and slow to heal. Although uncertain about the outcomes of their treatment, they still expected a speedy recovery. The main concerns of patients were for faster healing and pain relief, but they also expressed uncertainty about new technologies and worried about potential financial risks, as shown in Figure 4.

*“Feeling uncertain about the effectiveness of this method of treatment and worried that the treatment wouldn't be effective, I was really worried about becoming an experiment (test subject) when I was told by the doctor how good this method was and that I needed to sign a consent form to be treated.” (P5)*

*“Suffering from wounds for a long time, tried so many methods, spent a lot of money, but not heal completely, kept coming back..., even wanted to stop treating them and let them heal on their own. My daughter heard about a new treatment and asked me to try it. Although I hoped that this treatment would produce miraculous results, I was deeply concerned that it would still be a waste of money and would not be able to heal the wound completely.” (P10)[sic]*

### 3.2 Dual-dimensional problems during treatment

#### 3.2.1 Physical discomfort

Patients may experience discomfort during PRF therapy, but most of them tolerate it in anticipation of the outcome of the treatment, as shown in Figure 4.

*“I am sensitive to pain myself and usually have to rely on painkillers. Every time I thought about having to clean the wound, the anxiety started the night before. The pain was excruciating when the wound was cleaned, and there was nothing I could do but endure it.” (P2)*

*“When put the gel on the wound, it hurt a bit..., feel a strange feeling there, but duration is not long.” (P5, P11)[sic]*

*“Can't move too much after the treatment for fear of sweating and oedema, it's not very comfortable to move around, and afraid might accidentally touch the wound, affect the effect of the treatment.” (P9)*

*“Uncomfortable to take a bath, sometimes it's too hot, but don't dare wash it for fear of infection, only symbolically wipe it with a towel.” (P7)*

*“The wounds are bandaged for a long time, and the bandage is changed once a week. The wounds itch, but I don't dare scratch them, the feeling is too unbearable.” (P8, P10)[sic]*

#### 3.2.2 Process anxiety: cumbersome procedures

**Figure 5:**
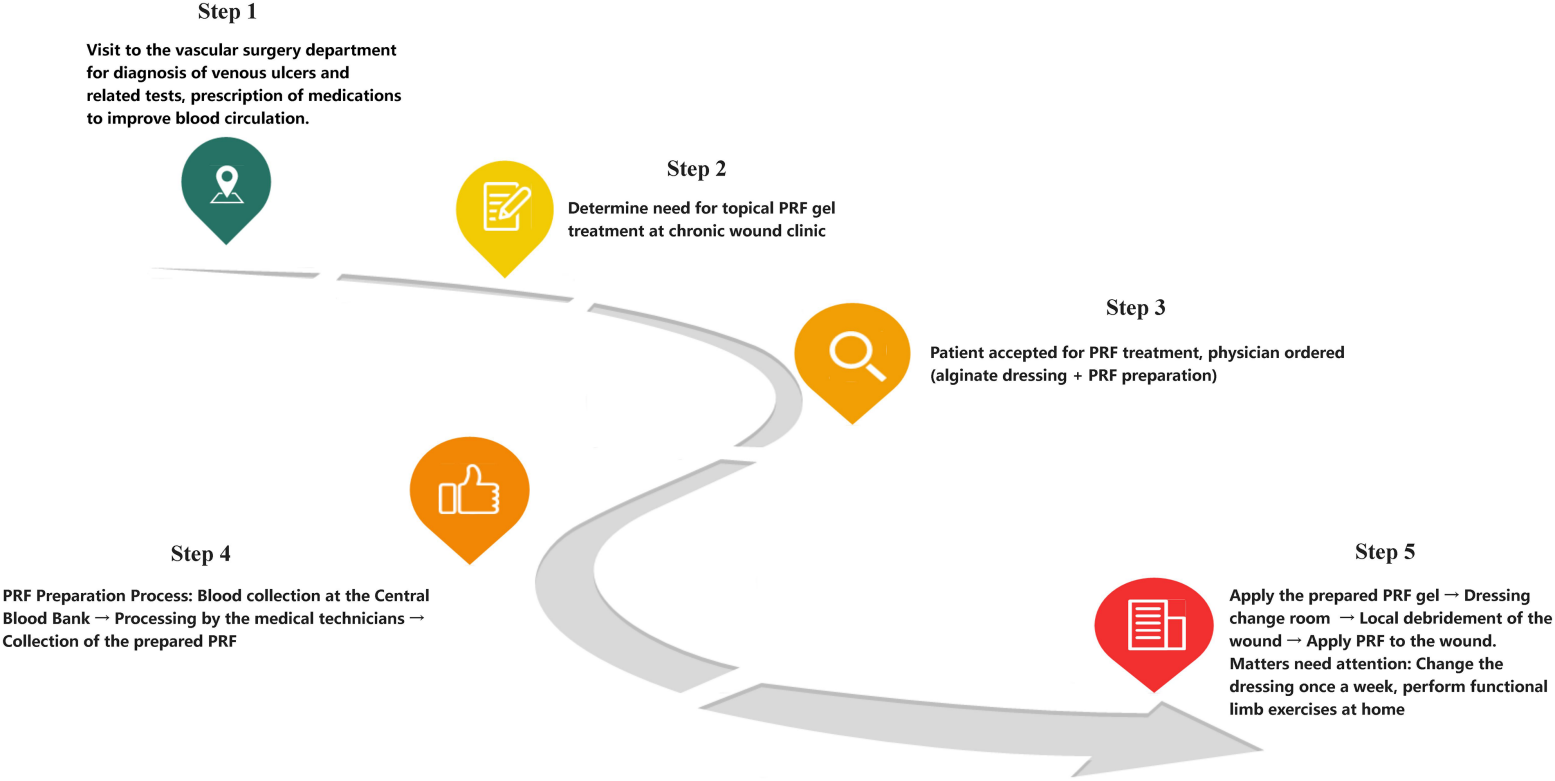
PRF gel treatment workflow for venous leg ulcers. This clinical pathway diagram illustrates the standard operating procedure for PRF treatment, highlighting steps that contribute to the patient-reported “cumbersome processes”: the segmented workflow between step 4 (blood processing at a distant laboratory) and step 5 (clinic application) forces vulnerable patients to transfer 3 + locations per session to validate the reports of “treatment exhaustion”.

Within the workflow of our hospital, the process involving travel between the blood bank and the wound clinic (blood collection–processing–waiting in line) was reported as cumbersome by some patients, particularly those with mobility impairments, as illustrated in Figure 5.

*“Treatment process is quite fast, but the preparation process, the need for blood collection, the processing, the need for queuing if there are a lot of patients, if you are in a hurry, it is more cumbersome, so people are a little bit anxious.” (P1)*

*“Long way from the medication room to the place where the blood is taken, I am old and have mobility problems, it will take me a long time to walk there. Every time I need my son to accompany me, I have to use a wheelchair, which means it will take up my family's time.” (P10)*

#### 3.2.3 Adaptive behavioral development

Under the guidance of doctors and nurses, patients gradually become more acclimatized, learn to deal with discomfort, and improve their wound management within 2 weeks.

*“Elevated legs above your heart (misinterpreting what the doctor meant and thinking that elevation would do the trick led to poor results, lots of oozing and swollen legs). Eat more protein- and vitamin-rich foods. Try to avoid external pressure or rubbing of the wound.” (P5)*

*“Ankle exercises, padded toes, moisturising around the wound should be done, the itchy edges of the wound can be relieved by distraction (cold compresses around the wound, gentle stroking, etc) and must not be scratched or rubbed locally, this is a lesson in tears (laughs) and try to avoid secondary injury to the wounds, the 'new skin' is too fragile.” (P11)*

### 3.3 Therapeutic effectiveness-driven biopsychosocial change

#### 3.3.1 Improvements in physiology and quality of life

**Figure 6:**
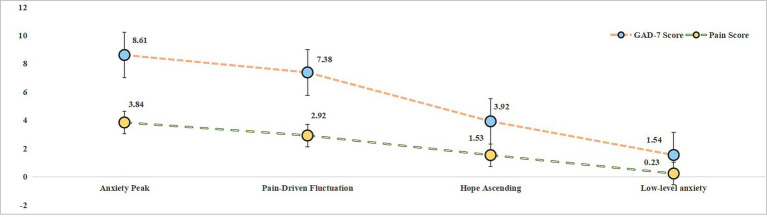
Dynamic emotional trajectory in VLU patients during treatment with PRF. Quantified psychological progression using GAD-7 anxiety disorder screening scale (0–21) and VAS pain rating scale (0–10). This longitudinal emotion mapping validates patient-reported positive transformations through quantifiable psychological shifts: The sustained descent from anxiety peak (8.61) to low-level anxiety (1.54) objectively mirrors the patient’s narrative of a change from anxiety to optimism.

Once PRF treatment was initiated, patients reported that the initial frequent tingling sensation was effectively relieved or even eliminated. The wound area gradually reduced, dressing changes were reduced to once a week, and patients regained greater freedom of movement. Many also reported a perceived reduction in financial burden compared to previous treatments, describing the therapy as ‘more cost-effective.’ These important changes contributed to patient satisfaction and appreciation of the treatment’s effectiveness, as shown in Figure 6.

*“With just one treatment with PRF, the pain disappeared, which makes me happy and look forward to getting better results later.” (P1)*

*“Surprised by the results of the treatment (laughs)... Really happy that the wound healed faster than I expected. Feeling of renewed hope in life.” (P4)*

*“Instead of having to change the dressing as often as used to (2-3 times a week), now only have to change it once a week... no longer restricted by the pain caused by the wound, have more time for my daily activities. The cost of the treatment is also much lower than the traditional method, reducing financial burden and making it cost-effective!” (P6) [sic]*

#### 3.3.2 Re-orientation and re-confidence

After treatment with PRF, patients shifted from anxiety to optimism, regained hope for life, restored self-confidence, and returned to a normal daily life. Their mobility improved significantly, and although regular monitoring remained necessary, they no longer needed frequent medication adjustments and had more time to engage in daily life, as shown in Figure 6.

*“Now that the wound is starting to heal, I'm starting to get more active, life has become more regular... before always worried about the wound, but now that I see that there is hope for a cure, I feel that the whole person is much more relaxed, and my mood much better than I did before.” (P5) [sic]*

*“Before the treatment, I was afraid that the doctor was experimenting on me, but after the PRF treatment, the wound was getting smaller and smaller, my mood was getting better and better, so doubts disappeared. They (doctors and nurses) were very professional and gave me a lot of psychological support, which made me feel at ease.” (P8)*

### 3.4 Dynamic psychological trajectories

#### 3.4.1 Anxiety-hope oscillation

**Figure 7:**
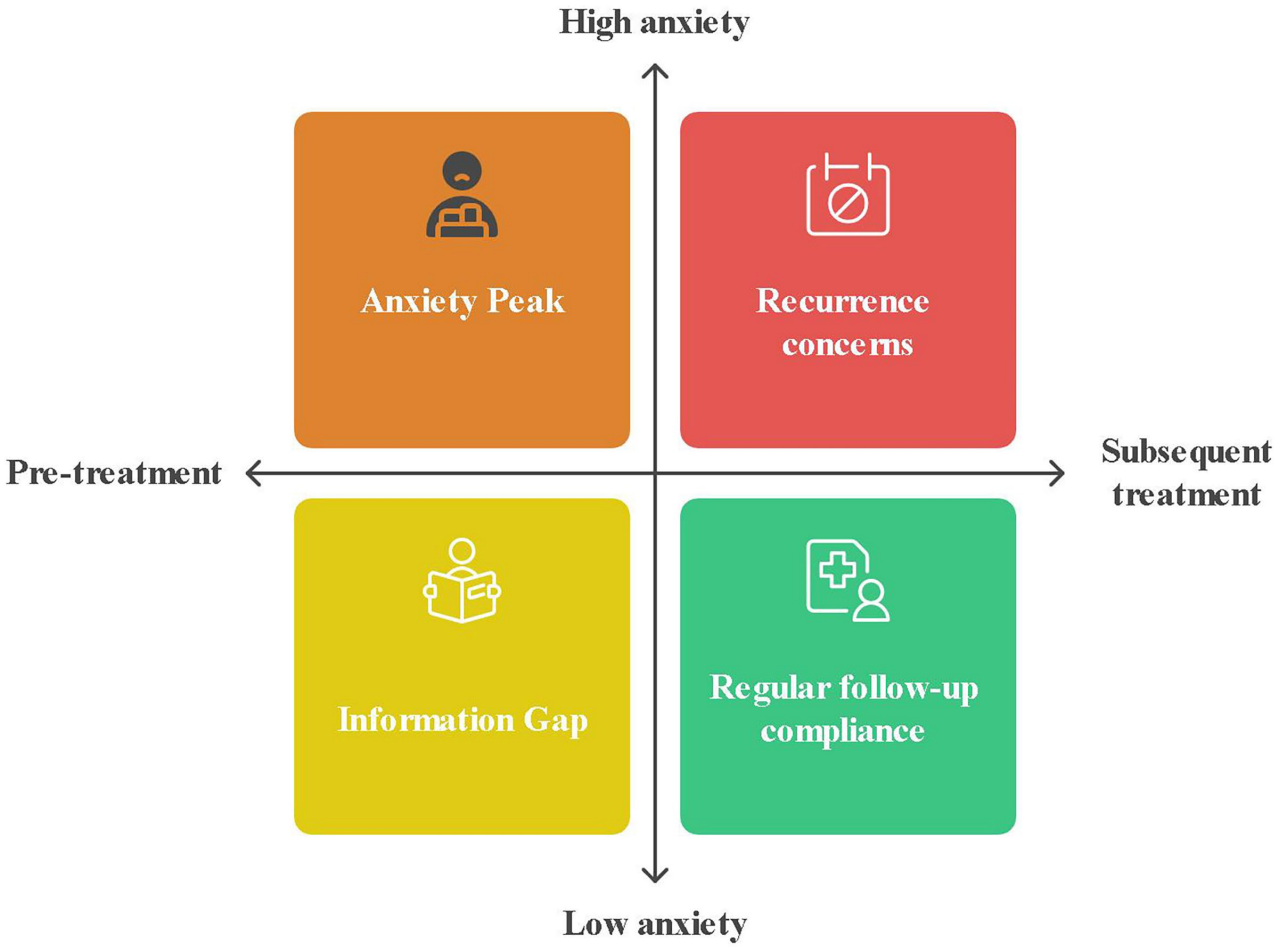
Quadrant plot of anxiety fluctuation dynamics driven by clinical feedback and recurrence fear. This model explains the development of anxiety in VLU patients through clinically relevant stages: the sustained low anxiety (post-treatment) with recurrence shadowing directly reflects the patient experience of decreased but still present anxiety. Clinician opinions dominated early anxiety fluctuations. The clinician’s assessment serves as a key trigger for the anxiety switch, and the patient reports that the doctor’s praise made him feel better, whereas the frown sent him into a depression.

At the beginning of treatment, patients were often anxious and concerned about both the effectiveness of the treatment and the side effects. Clinicians’ opinions about the dressing played a key role in influencing patients’ moods: positive feedback increased their sense of well-being, while unfavorable feedback contributed to feelings of depression. Although the wound gradually healed and anxiety decreased, the fear of recurrence remained a constant concern, as illustrated in Figure 7.

*“At the beginning of the treatment, I was particularly anxious, constantly worrying about whether the treatment would work or not, whether there would be any side effects(scar, aesthetic impact, infection...), and these thoughts made me anxious.” (P2)*

*“The mood can be described as up and down, and it can be decided when the doctor opens the bandage, if I say 'not bad, it's grown a lot', my mood is reassuring and a smile comes to my face; but if I say 'ah, how could this happen, it shouldn't be, didn't you do a good job..., the mood at that moment would be very low, just like a rollercoaster, falling from good expectations to a bad end..., may not be able to sleep as well at night, and any activity related to this is so cautious that it's actually quite hard on the psyche.” (P10)*

*“Every time you go to the hospital for treatment, the heart is very anxious, like opening a blind box, not knowing what will happen this time, worried about the recurrence of the disease and the increase in financial expenditure..., When the wound is getting better, anxiety is slowly decreasing, sleeping better, try to keep myself in a positive frame of mind.” (P6)*

*“Although the wound is slowly getting better and the anxiety is much less, the wound used to come back all the time, and now I can't stop worrying about it; I'm afraid it will come back, and my heart is always unsettled.” (P9)*

*"Deeply concerned about scar formation and cosmetic outcomes (particularly for summer attire), after witnessing another patient’s extensive, erythematous leg scar..., Although my wound is healing, persistent discomfort during showers, accompanied by anxiety about potential wound breakdown or recurrent infection, or recurrence... contribute to ongoing psychological distress.” (P3)*

### 3.5 Notable variations in the journey

*While core themes were shared, experiences varied. Younger patients (e.g., P6, 33y) expressed more frustration with activity restriction, whereas older patients (e.g., P10, 87y) emphasized dependence on family for logistics. Patients with larger ulcers (>20 cm^2^, P1, P9) described more intense initial anxiety about efficacy. Those with a history of multiple recurrences (P1, P3, P9) exhibited a more persistent ‘fear of recurrence’ even during healing.*

## 4 Discussion

This study used an innovative framework of mixed phenomenology and patient journey mapping to provide a comprehensive analysis of the multidimensional experience of patients with active VLU during treatment with PRF gel. Four major dimensions have been identified: pre-treatment cognitive dissonance with multidimensional expectations, dual-dimensional problems (physical and procedural discomfort), biopsychosocial shifts driven by efficacy, and dynamic psychological trajectories (anxiety–hope oscillation). PRF treatment not only improves wound healing but also improves psychological resilience through bioactive and psychosocial mechanisms, while information asymmetry, financial pressure, and communication between clinicians and patients remain critical factors in the patient experience. This study provided key evidence for optimizing the PRF gel treatment plan and improving patient care by clarifying the key cognitive conflicts, emotional dynamics, and key decision-making nodes in each stage.

### 4.1 Efficacy of PRF gel: bridging biological results and patient-centered assessment

Several previous studies have confirmed the beneficial effects of PRF gel in the treatment of wounds (21, 23, 25, 43). Chen et al. (24) conducted a randomized controlled study, which showed that venous leg ulcer (VLU) patients treated with PRF gel had significantly faster wound healing than patients treated with conventional therapy. These findings are consistent with our observations of the effectiveness of PRF gel in speeding up wound closure. These studies strongly confirm the clinical benefits of PRF gel in wound healing and provide a solid basis for our investigation.

However, going beyond these clinical metrics, our study examines in a unique way the interaction between patient expectations and perception of the rate of healing. During the psychosomatic phase of transition, patients experienced increased confidence as healing progressed beyond their expected time-to-effect, a psychological dimension that had been overlooked in previous studies, which focused exclusively on objective results (such as wound area reduction and healing time). While these metrics accurately quantify the therapeutic effect, they neglect the impact of subjective patient experience on adherence. Differences between perceived and actual rates of healing can cause anxiety or disappointment and potentially compromise compliance. Our study addresses this gap and provides a comprehensive framework for evaluating PRF treatment by integrating patient-reported outcomes.

Aside from the speed of healing, the quality of wound healing is another critical therapeutic endpoint. Studies indicate that growth factors in PRF gel increase the synthesis and remodeling of the extracellular matrix, promoting the formation of healthier scar tissue (44, 45). However, patient-centered assessments of scar esthetics, texture, and their psychosocial implications remain unexplored. Through in-depth interviews, we have found that patients prioritize not only wound closure but also the characteristics of post-traumatic scarring. Concerns about scarring, in particular its effects on esthetics and function of the limbs, persisted after clinical healing, underlining the need to integrate the reported quality of the treatment into the optimization of the treatment.

Regarding infection control, the intrinsic antimicrobial properties of PRF gel may reduce the risk of infections (46). However, the perception and concerns of patients about infection during treatment have not been fully investigated. Our findings reveal increased patient vigilance toward infections, where minor discomfort often triggers excessive anxiety, which adversely affects quality of life and adherence to treatment. Future studies should explore strategies to improve patient education on risk reduction and to reduce unnecessary psychological burdens.

It is critical to interpret these findings within the study’s methodological framework. The reported improvements in psychological wellbeing, such as reduced anxiety and increased confidence, are patients’ subjective perceptions. They likely result from a multifactorial interaction encompassing the potential biological effect of PRF, the structured clinical care received, positive clinician–patient communication, and the patients’ own evolving expectations and hope as the wound heals. Therefore, these psychological shifts cannot be isolated as a direct or sole consequence of PRF’s biological properties but represent the holistic outcome of the therapeutic journey.

### 4.2 Phased psychological dynamics of PRF therapy: from conflict to empowerment

Previous research on VLU has focused mainly on the general experience of patients, including quality of life, self-management practices, and treatment attitudes (8, 17). However, psychological research in patients undergoing PRF treatment remains limited. Using qualitative methodology, this study reveals nuanced psychological states specific to PRF treatment, enhancing understanding of patients’ psychological preparedness for PRF treatment, a topic that has been underexplored in the literature.

During the cognitive dissonance phase, patients experienced dissonance between information gaps about PRF treatment and their expectations, leading to distress. This finding adds a depth of understanding of the psychological state of patients before treatment that has not been fully addressed in previous studies. Limited access to comprehensible information, primarily relying on clinician explanations and institutional brochures, resulted in a superficial understanding (of principles, processes, and impacts) and a lack of certainty. This information asymmetry not only makes decisions difficult but also hinders adherence. Previous studies have acknowledged the concerns of patients, but they have not explored the root causes and consequences of cognitive dissonance unique to novel therapies such as PRF.

Regarding the influence of patients on treatment decisions, previous studies have not included a systematic analysis of patient decision-making patterns and determinants during PRF therapy (47). Our study clearly showed that at the crucial decision-making node of acceptance of PRF treatment, the economic burden is a key barrier that leads patients to delay treatment. This provides actionable insights for targeted interventions—a gap in previous research. In addition to financial constraints, our study identifies clinician trust and availability of treatment as key determinants of uptake. Skepticism toward clinical expertise or logistical hurdles (e.g., complex protocols and transportation challenges) may deter patients from pursuing PRF therapy. These findings highlight the need to increase patient confidence and streamline treatment workflow to improve acceptance of medication.

Previous studies have focused mostly on the overall emotional state of patients and rarely refined emotional changes at specific treatment stages (48). Our study reveals a fluctuating emotional trajectory: intense negativity during the two-dimensional challenge phase (which is characterized by physical pain and logistical obstacles) is followed by increasing hope during the psychosomatic transition phase as the healing progresses. This stepwise emotional mapping allows clinicians to deliver targeted interventions, such as improved pain management and empathic communication during the stress phase, combined with empowerment strategies to strengthen positive behavior and emotions during the recovery phase. The described anxiety-hope oscillation and reports of reduced distress represent patients’ subjective experiences and perceptions within the treatment context. These findings should not be interpreted as evidence that PRF gel therapy treats clinical anxiety or depressive disorders.

### 4.3 Self-management in PRF treatment: interaction between cognition, self-efficacy, and social support

Previous studies of PRF treatment have not adequately addressed the interaction between psychological cognition and self-control behavior, in particular, the role of psychological factors. From the perspective of social cognitive theory, self-efficacy, defined as the belief in the ability of an individual to perform the actions necessary to achieve the desired result, is a critical mediator between psychological cognition and self-control (49). Patients who have a positive perception of PRF therapy, such as confidence in their ability to facilitate healing through adherence and self-care, show increased self-efficacy, which motivates proactive self-management behavior. Conversely, low self-efficacy resulting from therapeutic pessimism or perceived ineffectiveness is correlated with passive or inconsistent self-control. In our study, patients who witnessed peers achieving positive results with PRF treatment reported increased self-efficacy, which was reflected in a higher adherence to wound-care protocols. By contrast, those with a longer disease burden and entrenched skepticism showed reduced self-efficacy and disengagement from tasks of self-management.

In addition, psychological cognition interacts synergistically with social support systems to shape self-control behavior (50). Strong social support, including family support, peer support, and clinician counseling, reinforces patients’ confidence in the effectiveness of treatment and increases psychological resilience, promoting a permanent commitment to self-management (51–53). Our findings suggest that patients who received attentive family support during PRF therapy were more likely to adhere to their own-care routines and attribute this adherence to a perceived feeling of emotional security and support. On the contrary, lack of social support has exacerbated feelings of isolation and helplessness, undermining psychological wellbeing and adherence to self-management.

These findings highlight the complex two-way relationship between psychological cognition and self-control in PRF-treated VLUs. Future research should prioritize interventions that address this nexus to optimize treatment outcomes. For example, structured health education programs could improve patients’ understanding of the mechanisms of the PRF and realistic expectations, thus increasing their self-efficacy. At the same time, the integration of psychological support (e.g., cognitive behavioral strategies) into clinical workflows can reduce therapeutic pessimism. In addition, optimizing social support networks through caregiver training or peer mentoring programs could strengthen the positive feedback loop between psychological resilience and self-sufficiency. Such multidimensional approaches have the potential to improve both biological healing and the quality of life of patients.

### 4.4 Limitations

This study has several limitations that should be considered when interpreting the findings. First, the sample size of 13 participants, although sufficient to achieve thematic saturation for the core research questions, is relatively small and derived from a single tertiary hospital in China. This limits the generalizability of the psychological journey model to broader populations, including those from different geographical regions, healthcare systems, or with more extreme clinical or social characteristics. Furthermore, the small sample precludes robust subgroup analysis to explore how experiences might vary systematically by factors such as age, ulcer severity, or comorbidities.

Second, while rich in experiential depth, the findings cannot establish causality. The reported associations between PRF therapy and improved psychological wellbeing are based on patient perceptions and temporal correlations; they do not permit the conclusion that PRF gel itself directly causes psychological benefits. A controlled quantitative or mixed-methods design would be required to disentangle the specific effects of the biological intervention from the contextual influences of professional care, patient expectations, and the natural healing process.

Third, methodological and contextual specificities affect the transferability and dependability of the findings. The patient journey map and the identified procedural barriers (e.g., inter-departmental coordination) are context-specific to the studied hospital’s workflow. The dual role of the researchers as clinicians and interviewers, despite mitigation through reflective journaling and involving a non-clinical analyst, introduces potential for social desirability bias and interpretive bias. Although consensus coding was used, the lack of formal inter-coder reliability statistics may affect the dependability of the coding framework. Additionally, the assessment of psychological distress relied on self-report and the GAD-7 screening tool, not on standardized diagnostic interviews.

Fourth, there was no formal health-economic evaluation, so discussions of cost are confined to patient-reported perceptions of burden. The study duration was also insufficient to evaluate long-term PRF outcomes, healing maintenance, or the psychological dynamics related to ulcer recurrence.

Future research should address these limitations by employing larger, more diverse cohorts, integrating longitudinal physiological and validated psychiatric measures, conducting formal cost-effectiveness analyses, and extending the follow-up period to fully investigate the long-term biopsychosocial effects of PRF treatment.

## 5 Conclusion

This study employed an integrated approach of patient journey mapping and qualitative analysis to systematically map the multidimensional experiences and document the dynamic psychological trajectories of patients with VLU undergoing PRF gel therapy. The findings illustrate that within the context of PRF therapy, patients reported not only perceived enhancements in wound healing but also a reduction in anxiety and an increase in trust toward the treatment. This positive shift from initial skepticism to confidence was closely associated with supportive clinician–patient communication and psychosocial care integrated into the treatment process. However, critical barriers such as information gaps, financial burdens, and procedural complexity were identified as persistent challenges to adherence and the overall care experience. The analysis further delineated a dynamic oscillation between anxiety and hope throughout the treatment journey, highlighting clinician communication as a pivotal factor influencing patients’ reported psychological adaptation.

To address these identified challenges, the study proposes holistic strategies including enhanced patient education, streamlined clinical workflows, and strengthened multidisciplinary support. These recommendations aim to optimize the delivery of PRF therapy and the broader care context, thereby potentially improving its integrated bio-psychosocial effectiveness. Collectively, these insights offer a foundational, patient-centered evidence base for refining personalized care approaches and clinical protocols in the management of chronic wounds.

## Data Availability

The original contributions presented in the study are included in the article/Supplementary material, further inquiries can be directed to the corresponding author.
