# Supplementary material for: A patient journey mapping study of lived experiences during platelet-rich fibrin gel therapy for venous leg ulcers
Source: Front Med (Lausanne). 2026 Jan 9;12:1652687. doi: 10.3389/fmed.2025.1652687 (PMC12827078; doi:10.3389/fmed.2025.1652687)
Supplement: Supplementary file 1 [file Supplementary_file_1.pdf]

Supplementary Table 1 Consolidated criteria for reporting qualitative studies (COREQ): 32-item checklist

No	Item	Guide questions/description	
**Domain 1 Research team and reflexivity**			
Personal Characteristics			
1	Interviewer/facilitator	Which author/s conducted the interview or focus group?	Fei fei Cui, Rongting Wang, Shuainan Chen, Qiaodan Hu
2	Credentials	What were the researcher's credentials? *E.g. PhD, MD*	Master of Nursing (FFC); Bachelor of Nursing (RTW, SNC, QDH)
3	Occupation	What was their occupation at the time of the study?	Nurse
4	Gender	Was the researcher male or female?	Male (FFC); female (RTW, SNC, QDH)
5	Experience and training	What experience or training did the researcher have?	-This study was conducted by Feifei Cui, who has a master's degree in nursing, and who has extensive academic background and professional experience in the field of trauma, such as pressure ulcers, and lower extremity ulcers. The researcher received systematic training in qualitative research methodology, including completing the strategies and considerations of nursing mixed methods or qualitative research methods, and received a certificate related to evidence-based trainer at Dongyang Hospital of Wenzhou Medical University.<br>-In the past five years, the researcher had participated in several qualitative research projects, such as coping with ulcer-inducing symptoms in rural patients with lower extremity venous ulcers inner experience; Stress overload, influencing factors, and psychological

			experiences of nurse managers and published papers. He has the ability to conduct in-depth interviews, observations, and research. He has skills in in-depth interviewing, observation, and textual analysis, and has extensive experience in data analysis and critical thinking.<br>-All members of this research team were actively involved in continuous professional development activities, regularly attend academic conferences, and read the latest research literature to keep their knowledge and skills up to date. In addition, they have a strong foundation in teaching nursing ethics, have assisted in the completion of a training program on the application of the “Four Themes Approach” to phased training in enhancing the ethical practice of new nurses, and have demonstrated sensitivity and responsibility to ethical issues in several research projects.<br>-Rongting Wang, Shuainan Chen, Qiaodan Hu were responsible for conducting the interviews and preliminary analysis of the data in this research project, and worked closely with other team members to ensure the smooth progress of the study.
Relationship with participants			
6	Relationship established	Was a relationship established prior to study commencement?	-Prior to the commencement of this study, preexisting relationships existed between the researcher and the patients of venous leg ulcer.<br>-These relationships were primarily established through the following channels: patients with lower limb venous ulcers visit the wound treatment clinic for wound care, education on related knowledge, and referrals from other chronic wound patients.
7	Participant knowledge of the interviewer	What did the participants know about the researcher? e.g. personal goals, reasons for doing the research	-In this study, the patients of VLU were fully informed of the researcher's background information prior to the start of the study. The researcher clearly communicated the purpose of the study and the reasons for conducting this study to ensure that participants had a clear understanding of the study.<br>-The importance of informed consent from the patients of VLU was also emphasized and it was ensured that they understood the content and

			process of the study, and also informed about how their data would be handled by the researcher and how their privacy and confidentiality would be protected.
8	Interviewer characteristics	What characteristics were reported about the interviewer /facilitator? e.g. Bias, assumptions, reasons and interests in the research topic	-The interviewers for this study were trained in qualitative research, had an in-depth knowledge of phenomenological methods, and had extensive practical experience in their teaching of ethics.<br>-The interviewers selected for this study were very interested in the research topic, and were committed to exploring new insights in the field. Their ability to demonstrate a high level of communication skills and trust building in their interactions with participants helped to create an environment of open and honest dialog.<br>-Interviewers during the study minimized the impact of personal bias through self-reflection and discussion with colleagues.
**Domain 2**<br>**Study design**			
Theoretical framework			
9	Methodological orientation and Theory	What methodological orientation was stated to underpin the study? *e.g. grounded theory, discourse analysis, ethnography, phenomenology, content analysis*	Phenomenology focuses specifically on the lived experiences of individuals and how these experiences constitute their understanding of the world. Phenomenology was chosen as the methodological basis for this study, combined with patient journey maps, because we wanted to explore the inner reactions experienced by venous ulcer patients undergoing new autologous blood treatment methods, such as PRF, and the emotional fluctuations they experienced at different stages of treatment.<br>**Page 5, Line 156-157**
Participant selection			
10	Sampling	How were participants selected? e.g. purposive, convenience, consecutive,snowball	-In this study, we used purposive sampling to select. We set clear inclusion criteria to ensure that participants were representative of the specific population we were studying. **Page 5, Line 129**

11	Method of approach	How were participants approached? e.g. face-to-face, telephone, mail, email	In this study, face-to-face interviews were conducted<br>**Page 6, Line 197**
12	Sample size	How many participants were in the study?	13 Participants<br>**Page 5, Line 144**
13	Non-participation	How many people refused to participate or dropped out? Reasons?	-During the recruitment process for this study, four patients refused to participate. The main reasons for choosing not to participate in the interview were the long distance, inconvenient transportation, language barriers, and lack of family accompaniment. All participated in the interviews during the conduct of the study and no one withdrew from the study.<br>-Strict ethical guidelines were adhered to throughout the study to ensure that all participants were aware of their right to withdraw from the study at any time without suffering any adverse consequences.
Setting			
14	Setting of data collection	Where was the data collected? e.g. home, clinic, workplace	-The interview site for this study was chosen to be in the outpatient clinic consultation room, which is beautiful, secluded, and comfortable environment that helps to promote open and honest dialog.<br>**Page 6, Line 197-198**
15	Presence of non-participants	Was anyone else present besides the participants and researchers?	no
16	Description of sample	What are the important characteristics of the sample? e.g. demographic data, date	-We selected suitable venous ulcer patients based on the inclusion and exclusion criteria, and enrolled the final participants based on sample saturation. The general information of the enrolled venous ulcer patients is detailed in ***Table 1,2***.<br>**Page 9, Line 305-316**
Data collection			
17	Interview guide	Were questions, prompts, guides provided by the authors? Was it pilot tested?	-In this study, developed an interview guide based on literature review, consultation of experts (three vascular surgeons, two wound specialists) and pilot interviews (n=3).

			-To ensure the validity of these questions, the interview outline was critically reviewed and three patients were selected for pretesting before the study began. The purpose of the pretest was to test comprehension of the questions, assess the feasibility of the data collection process, and collect feedback from the participants to revise the questions based on the feedback.<br>**Page 6, Line 188-189**
18	Repeat interviews	Were repeat interviews carried out? If yes, how many?	no
19	Audio/visual recording	Did the research use audio or visual recording to collect the data?	-In this study, we used audio recordings to collect data. This method allowed us to accurately capture what the participants were saying and the audio recording data was transcribed into text and used for data analysis along with field notes and other data to ensure accuracy. The transcription process was completed by trained transcriptionists.<br>-Before the recording began, all participants were clearly informed of the purpose of the recording and their informed consent was obtained. We emphasized our commitment to protecting their privacy and explained how the audio recording data would be securely stored and used.<br>**Page 6, Line 199**
20	Field notes	Were field notes made during and/or after the interview or focus group?	-The interviewer was responsible for taking notes in real time during the interviews in this study.<br>-Notes included, but were not limited to, body language, facial expressions, changes in tone of voice, and observations of participants' emotional states. These notes allowed for the capture of details that may have been missed when reviewing the recordings.<br>**Page 6, Line 200**
21	Duration	What was the duration of the interviews or focus group?	**Page 6, Line 198**
22	Data saturation	Was data saturation discussed?	Yes. **Page 5, Line141-143**
23	Transcripts returned	Were transcripts returned to participants for comment and /or correction?	-The group members in this study established a WeChat group, where the interviewer compiled an electronic version of the transcribed text and returned it to the participants for them to comment and/or make

			corrections.<br>- Considering that some patients may not be able to access WeChat or have difficulty reading text, and therefore cannot use electronic devices, we will conduct on-site paper-based text confirmation when patients return for follow-up visits.<br>-In order to validate the accuracy of the data and to ensure that the data analysis was based on the most authentic and comprehensive participant experiences, participants were encouraged to suggest corrections to any inaccurate or incomplete sections, their feedback was taken into serious consideration, and the transcribed text was corrected accordingly.<br>-This process was clearly explained to participants before the study began and their informed consent was obtained.<br>**Page 7, Line 217-222**
**Domain 3**<br>**Analysis and findings**			
Data analysis			
24	Number of data coders	How many data coders coded the data?	-There were three coders involved in the data coding process in this study. The coders were trained on the research questions and coding techniques before they started coding.<br>-The coding process was conducted through independent coding, and when disagreements were encountered, they were encouraged to engage in open discussion and share ideas with the rest of the team in order to reach a consensus, aiming to improve the accuracy of the coding and to ensure that all relevant themes were fully considered.<br>-This study did not use coding tool software, but only manual coding. After integrating all interview text data, a total of 173 codes were generated.

25	Description of the coding tree	Did authors provide a description of the coding tree?	No<br>-This table illustrates the progression from representative meaning units (direct quotes) to sub-themes and finally to the overarching themes reported in the study (e.g., showing how the quote "I was worried about becoming an experiment…" contributed to the sub-theme "Internal struggle between expectations and concerns" under the theme "Cognitive dissonance").
26	Derivation of themes	Were themes identified in advance or derived from the data?	-After in-depth analysis of the interview data, themes were generated using Colaizzi's (1978) method of data analysis. These themes were derived directly from the participants' narratives and reflected their experiences and perspectives.<br>-The process of distilling new themes was conducted through a combination of participant feedback and team discussion.
27	Software	What software, if applicable, was used to manage the data?	Not applicable
28	Participant checking	Did participants provide feedback on the findings?	-The WeChat group was established at the beginning of the study, and the researcher provided an electronic copy of the transcribed text, a summary of the study's main findings and themes to the participants through the WeChat group format, allowing them 1 week to review and comment on the preliminary results to ensure that the findings accurately reflected the reality of what they experienced in their clinical practice.<br>-Considering that some patients may not have access to WeChat or may have difficulty reading text, and therefore cannot use electronic devices, we will conduct on-site paper-based text confirmation during patient follow-ups. If patients have any questions or additions regarding the text, we will promptly make modifications based on their feedback.<br>-We emphasized the importance of their comments to improve the credibility and relevance of the study, and received some of the valuable feedback that helped to further validate and refine our conclusions.
Reporting			
29	Quotations presented	Were participant quotations	-In the results section of this study, we used direct quotes from

		presented to illustrate the themes / findings? Was each quotation identified? e.g. participant number	participants to vividly illustrate our themes and key findings. These quotes provide rich evidence for our analysis, helping readers gain a deeper understanding of the real feelings experienced by patients with venous leg ulcers during treatment with autologous blood PRF.<br>-We selected citations to demonstrate the diversity and depth in the data while ensuring that they accurately reflect the themes derived from the data.<br>-Each citation was identified through a unique participant number to protect participant privacy.
30	Data and findings consistent	Was there consistency between the data presented and the findings?	Data segments were carefully selected to reflect the theme of the study and were presented in detail in the findings section, which ensured a high degree of consistency between the data presentation and the findings of the study
31	Clarity of major themes	Were major themes clearly presented in the findings?	-The major themes have been clearly and unambiguously presented in the results section, subheadings and clear paragraphs have been used to differentiate the different themes.<br>-Themes have been presented through detailed textual descriptions and diagrams to help readers understand better.<br>**Figure 2 and figure 3**<br>**See the results section of the article**
32	Clarity of minor themes	Is there a description of diverse cases or discussion of minor themes?	In addition to the major themes, we also provided descriptions of secondary themes. The descriptions of the diversity cases showed how the research themes manifested themselves in different contexts and were able to provide a fuller picture of the participants' experiences. These secondary themes revealed other important patterns and insights in the data that help us understand the research questions more deeply.<br>**See the results and discussion section of the article**
